# Factors that influence management of postpartum hemorrhage among midwives in a rural setting in Kenya

**DOI:** 10.4314/ahs.v21i1.39

**Published:** 2021-03

**Authors:** Doris Mumbi Muthoni, Priscilla Njeri Kabue, Elizabeth Kurwa Ambani

**Affiliations:** Department of Community and Reproductive Health Nursing, Kenyatta University

**Keywords:** Midwifery factors, management of postpartum haemorrhage

## Abstract

**Background:**

Postpartum hemorrhage is the cumulative blood loss of 500 milliliters or more in a spontaneous vaginal delivery and approximately 1,000 milliliters or more for caesarean section birth and a leading cause of maternal mortality^1^.

**Objective:**

The overall aim of the study was to determine midwives' factors that influence the management of PPH.

**Methods:**

A descriptive cross-sectional study that employed a quantitative approach through the use of a research self- administered questionnaire and an observational checklist targeting midwives were used. 85 midwives filled the questionnaire and 71 were observed respectively. The study was hospital- based conducted in Muranga County, Kenya. Convenience sampling technique was used to select the midwives in the study sites.

**Results:**

The following factors were statistically significant in influencing management of postpartum hemorrhage; age (P-value = 0.021). professional qualification (P= 0.047), experience in management of PPH (P= 0.032) and training on emergency PPH (P= 0.010), knowledge factors that were found to influence the management of PPH positively include knowledge on; prevention of PPH (p value-0.000), correct use of prophylactic uterotonic agents (P= 0.000), uterotonics use (P= 0.043), uterine massage during 3^rd^ stage of labour (P= 0.012), examination of the placenta (P= 0.034), management of PPH (P= 0.028), causes and diagnosis of PPH (P= 0.001), (Fischer's exact value= 0.043).

**Conclusion:**

Results of the study indicate a statistical association between midwives' factors and management of PPH.

## Introduction

### Background of the Study

Postpartum hemorrhage (PPH) is the leading cause of maternal death yet the most preventable cause of maternal mortality. All women who carry a pregnancy beyond 20 weeks' gestation are at risk for PPH and its sequelae. As the leading cause of morbidity and mortality in childbirth, it occurs in approximately 1% to 6% of all deliveries 2, 3, 4.

Globally, maternal mortality is alarmingly decreasing, but in developing countries, especially Subharan Africa maternal death is caused by Hemorrhage due to infrastructure limitation, lack of skilled birth attendants, and inappropriate management of active third stage of labour. In sub-Saharan Africa, 25% of maternal deaths are as a result of hemorrhage and 15% of the bleeding occurs during the postpartum period 5. Kenya is ranked the eleventh country with a high maternal mortality rate (362 per 100,000) 6. In Murang'a County, the prevalence of PPH remains a challenge with a total of 20 maternal deaths due to complications of postpartum hemorrhage recorded in 5 years from 2014 to 2019 7.

Postpartum hemorrhage has been linked to some independent variables and intervening variables that influence its outcome in the health care setting. Midwife's profession and year of graduation are some important factors that associate with the knowledge towards active management of the third stage of labour. A study done in Tanzania highlighted professional qualification as an important factor in managing PPH 8. Active management of the third stage of labour is one strategy that is inexpensive and also known to be effective in the prevention of PPH and it involves the midwives carrying out three interrelated but independent processes: prophylactic administration of the uterotonic agent, controlled cord traction, and uterine massage 5. The use of oxytocin as a first-line drug by midwives helps prevent PPH 9.

The current scenario in developing countries mandates research on newer and practicable strategies to tackle PPH which can be implemented effectively and have an upper edge over the existing practices in the management of PPH 10. Hence, the study aimed to determine the knowledge and skills of midwives in the management of postpartum hemorrhage.

## Methods

A descriptive cross-sectional study was used to determine the influence of midwives' factors in the management of postpartum hemorrhage. The study was hospital-based conducted in Muranga County, Kenya. The study involved midwives who had worked in the maternity department for at least 6 months and provided consent to participate in the study. To select the midwives, a proportionate sampling strategy was used. One level five hospital and 6 randomly selected Sub-County hospitals were chosen for this study since they offer maternity services. The convenience sampling technique was used to select the midwives working in Murang'a County referral hospital and Sub-County hospitals since the focus was on those midwives that were conveniently available during the study period. Muranga county hospital was selected since it is the only County teaching and referral hospital and has a high workload in maternity services with high maternal mortality in the County. A well-designed and pretested questionnaire was used to validate the research instruments. The questionnaires were designed in relation to the conceptual framework and research objectives. The data was collected by the use of self-administered questionnaires and observations. The self-administered questionnaire featured both closed and open-ended questions and took each midwife who consented to participate an average of 10–15 minutes to fill. For the observation, a checklist was used to observe the actual skills that midwives employed during the second and third stage of labour and was filled by the researcher and the research assistant. On average each observation took 30 minutes during the second stage and third stage of labour. Statistical analysis using the Statistical Package for Social Sciences was done. Data were edited, coded, and entered. A descriptive analysis was done to determine percentages and means. Contingency tables were used to establish the extent of association between the variables. The results were presented by the use of graphs, pie charts, and tables. Approvals to conduct the study were given by Kenyatta University (KU) Ethics and Research Committee and the authority to undertake the study was sought from the National Council of Science and Technology (NACOSTI). Permission to carry out the research was sought from Murrang'a County Hospital administrator.

## Results

The objective of the study was to identify midwifery factors, that is knowledge and skills that influence PPH management. In total 85 midwives filled the questionnaire and 71 midwives were observed while conducting deliveries

### Socio-demographic characteristics

The majority of the respondents 88.2% (75) were female while 11.8% (10) were males. (36.5%) of the respondents were aged between 31 and 40 years followed by 20 to 30 years which accounted for 31.8% (27). The midwives aged between 51 to 60 years accounted for 5.9% (5). Majority 74.1% (63) of the midwives were registered community health nurses followed by enrolled community health nurses accounting for 15.2% (13). Only 10.6% (9) of the midwives had a degree in nursing qualification. Majority of 17(20.0%) of the midwives had worked in the labour ward for 4 years followed by those who had work experience of 5 years. The average work experience in the labor ward for all the midwives was 4.38 years with a standard deviation of 3.176. See table 1.

**Table 1:** Socio-demographic characteristics of the participants

Variable	Frequency	Percent
**Gender**		
Male	10	11.8
Female	75	88.2
**Age**		
20–30 years	27	31.8
31–40 years	31	36.5
41–50 years	22	25.9
51–60 years	5	5.9
**Profession qualification**		
ECHN-Enrolled community Health Nurse	13	15.2
KRCHN	63	74.1
BSCN-Bachelor of Science nursing	9	10.6
**Midwives labour ward experience**		
0–3years	32	37.7
4years	17	20.0
5 years -thirteen	31	36.4
Not specified	5	5.9

Notes: N = 85,

### Socio-demographic characteristics influencing the management of PPH

The association between gender and management of PPH was not statistically significant (X 2 = 1.133, df = 1, P-value= 0.092). There was a statistically significant association between age (X 2 = 4.815, df = 3, P-value = 0.021), professional qualification (X2 =8.817, df= 4, P-value of 0.047) and midwives experience (X2 =9.513, df=10, P-value=0.032) in the management of PPH.

### Midwives factors influencing the management of PPH

The respondents were requested to give details on the following factors;(1) training of emergency PPH, (2) knowledge on risk factors, prevention, and management, (3) Skills in PPH.

### Respondents Training on Emergency PPH

On midwives training on emergency PPH, the target was on three pieces of training (AMSTL, EmONC, and Respective maternity care) where the respondents were to indicate whether they have been trained. 31.8% (27) and 36.5% (31) of the midwives had trained on AMTSL and EmONC respectively. None of the midwives had been trained on ‘respective maternity care’.

### Respondents Knowledge on Prevention of PPH

When the respondents were asked about practices of PPH prevention, majority 96.5% (82) reported knowing that administration of uterotonic is used in the prevention of PPH. Likewise, 87.1% (74) and 88.2% (75) of the respondents reported that controlled cord traction (CCT) and uterine massage are effective methods of preventing PPH respectively. However, 1.2% (1), 7.1% (6), and 8.2% (75) reported no knowledge on uterotonic administration, controlled cord traction, and uterine massage respectively.

### Respondents' Knowledge on Management of PPH

Majority 34(40%) indicated that they would recognize PPH immediately after delivery by measuring the blood loss after delivery, 28(32.9%) by observing maternal vital signs, 18(21.2%) by looking at the soaked linen after delivery, and 5(5.9%) did not know.

### Relationship between Midwives knowledge Factors and Management of PPH

Midwives factors that were statistically significant in the management of PPH include; work experience in the labor ward (p-value of 0.032), knowledge on prevention of PPH (p-value=0.000), and knowledge on the management of PPH (p- value=0.028).

### Skills in handling PPH

The respondents were requested to indicate their responses concerning the handling of PPH on a 3-Point scale (3 = able to do without assistance, 2 = Able to do with assistance, and 1 = Not able to do even with assistance). Majority of the respondents responded to have the skills required in handling PPH where 75(88.2%) could identify the risk factors of PPH without assistance, 82 (96.4%) of the midwives could diagnose PPH without assistance, 78(91.8%) could suture perineal tears without assistance and 80(94.1%) suture episiotomy without assistance. About bimanual compression to the uterus, 41(48.2%) of all midwives indicated that they were able to do it without being assisted. However, 45.9% (39) of the midwives reported performing bimanual compression of the uterus with assistance, while 5.9% (5) did not have the skills to perform the bimanual expression.

For the predictors of Management of Postpartum Hemorrhage Logistic regression was performed to identify the socio-demographic characteristics, midwifery factors, that were significant predictors of the management of PPH. The results of the analysis are shown in table 4 with the adjusted odds ratio in table 5.

**Table 4:** Results of binary logistic regression for factors associated with the management of PPH

Factors		OR	P-value	95% CI lower	95% CI Upper
Gender	Male	Reference			
	Female	0.89	0.711	0.38	1.92

Age	20–30 years	Reference			
	31–40 years	1.62	0.229	0.88	2.14
	≥ 40 years	2.85	0.003	1.45	6.71

Professional qualification	Enrolled	Reference			
	Registered	1.64	0.231	0.52	2.99

Experience in maternity (years)	<5	Reference			
	≥5	3.04	0.000	2.62	6.01

Note: OR = Odd Ratio, CI = Confidence Interval

**Table 5:** Results of binary logistic regression for factors associated with Management of PPH with an adjusted odds ratio

Factors		AOR	P-value	95% CI lower	95% CI Upper
Age	20–30 years	Reference			
	31–40 years	0.87	0.028	0.11	1.75
	≥ 40 years	1.85	0.021	0.22	3.21
Experience in maternity (years)	<5	Reference			
	≥5	2.04	0.031	1.12	4.51

Note: AOR = Adjusted Odd Ratio, CI = Confidence Interval

### Respondents Utilization of Current Guideline in the Management of Postpartum Hemorrhage

This section was to show results of the analysis of data collected on the management of PPH using an observation checklist adopted from the Pathfinder International competence-based checklist. Areas covered on the checklist were: i) preparation of birth, ii) emotional support, iii) preparation of the midwife, iv) birth, v) AMSTL components (administration of a uterotonic drug, controlled cord traction (CCT), and uterine massage). All items in the sections were measured on a 3-point scale ranging where 1 indicated not performed, 2 indicated needs improvement, and 3 demonstrated the activity is competently performed. The scores for each section were added and the average for each score was calculated. The findings of the skills were summarized using the pathfinder international Midwives checklist result.

Pathfinder International Midwives Competent Checklist Result Using the Five Observed Areas

- Only 27.2% of the midwives had competently prepared for the birth.
- Only 18.3% were able to competently offer emotional support.
- Only 25.9% of the midwives prepared correctly for the procedure.
- Only 26.2% competently preparing for the second stage of labor.
- Only 35.5% of the midwives knew uterotonic drug administration.
- Only 25.9% of the midwives practiced skills of Controlled Cord Traction.
- Only 27.1% of the midwives knew when to perform the uterine massage

## Discussion

Performance bias was a limitation for respondents being observed during delivery and was addressed by hindering them from viewing the observational checklist.

### Socio-Demographic Characteristics

Age showed a statistical significance in the management of PPH while gender does not. This corresponds with a study done by Rosseau et al that reports that the age of midwives is significantly associated with the management of PPH 11. In this study majority of the respondents were females. These findings are similar to a study carried out by Zalalem et al that showed high respondents as females 5. There was a statistical significance between professional qualification and management of PPH. This corroborates with a study done in Tanzania that showed that professional qualification had a significant association with the management of PPH 8. This study showed a statistical significance between experience in the labour ward and the management of PPH. This is in line with a study by Adane et al in northern Ethiopia which showed similar findings 12.

### Midwives knowledge factors that influence the management of PPH

In this study, factors established to influence the management of PPH were assessed.

Only a few midwives had been trained on the 3 critical pieces of training (AMTSL, EmONC, and Respective Maternity care). The gap in training could be a result of resource constraints by the government, poor competence among providers, and also poor implementation of the guidelines. This is similar to a study done in South Ethiopia that reported a low number of midwives training on AMSTL 5.

The study findings revealed that the majority of the midwives knew about the prevention of PPH with the majority reporting the three practices (administration of the uterotonic drug, performing controlled cord traction, and uterine massage) as strategies of preventing PPH. This is similar to a study done in Nigeria that showed that the majority knew the use of the uterotonic drug in the prevention of PPH 9.

This finding is higher than that of a study done in Ethiopia with a few midwives reporting knowledge on the three interrelated practices to prevent PPH 5. Among those observed conducting deliveries, few midwives competently showed skills in the prevention of PPH by use of the three practices used. This is a result of the knowledge gap due to the lack of training updates.

On midwives' knowledge on the management of PPH, majority of the midwives reported oxytocin as the drug of choice in PPH management. These findings coincide with a study done in Nigeria that showed that the majority of the midwives reported the use of oxytocin as the drug of choice, as per WHO recommendation 9. On knowledge on the timing of administration of prophylactic uterotonic, majority of the midwives from the study had adequate knowledge that administration should be within a minute after delivery of the infant. On observed deliveries, few showed competent skills on the timing of the prophylactic uterotonic administration within a minute after delivery of the baby while the majority did not administer the uterotonic on the timing laid as per guidelines. This is similar to a study done in Adis Ababa, Ethiopia that showed that majority of the respondents gave the uterotonic within one minute after delivery of the infant 13. On knowledge on uterine massage, majority of the respondents agreed that they perform uterine massage, indicating that the timing to perform is during the third stage of labour after delivery of the placenta. This is similar to a study done by Nkwonta et al that showed that the majority of the midwives knew that practice on uterine massage is during the third stage of labour 14. Irrespective of the midwives reporting to know about uterine massage, few practiced the skill. This could be due to the knowledge gap and high workload.

Majority of the respondents reported to practically examine the placenta for completeness while few indicated that they do not examine the placenta. This is in an agreement with a study by Mutunga that showed that few midwives reported examining the placenta 15.

Only a few of the midwives from the study reported knowledge on recognition of PPH immediately after birth through the different measures that were used. This is similar to a study done by Hancock et al which indicates the majority of the midwives were highly inaccurate at estimating blood loss as a volume 16. From this study majority of the midwives were able to indicate the 4 T's that cause PPH. This coincides with a study done in Addis Ababa, Ethiopia where midwives reported uterine atony as the leading cause of PPH 13.

On the first response of a midwife on diagnosing PPH majority of the respondents reported shouting for help, followed by exploring the cause and later arrest of bleeding. Few nurses reported not know how to respond. This finding coincided with a study done in Kiambu, Kenya that showed that the majority of the midwives reported shouting for help after diagnosing PPH 15.

### Observation using the checklist

A high proportion of midwives on skill performance scored low as the majority were either ranged as needs improvement or not performed as per the 3-point scale on skill items like birth preparedness, actual delivery, and components of active management of the third stage of labour.

## Conclusion

Midwives' factors that influenced the management of PPH were; professional qualification, labour ward experience, training on emergencies, and midwife's knowledge and skills factors. Generally, the researcher found out that despite midwives' knowledge in the management of PPH, demonstration of skills was low.

The study recommends needing to emphasize training midwives on skills of PPH management to prevent postpartum hemorrhage

## Figures and Tables

**Table 2:** Respondents Knowledge Related Factors and Management of PPH

ITEM		X^2^ (df)	p-value
Information of the midwives on the training of emergency PPH	Chi-square	6.568	0.010
	df	1	
	N	85	
Midwives Knowledge on prevention of PPH	Chi-square	78.841	0.000
	df	1	
	N	85	
Knowledge on management of PPH	Chi-square	0.748	0.028
	df	2	
	N	85	

**Table 3:** Respondents Skills in Handling PPH and Management of PPH

Skills in handling PPH	Chi-square	(df)	p value
Identification of risk factors of PPH	X ^2^ = 8.458	(2)	*p = .015
Diagnosis of PPH	X ^2^ = 14.516	(2)	*p = .005
Bimanual compression to the uterus	X ^2^ = 2.619	(2)	*p = .042
Suturing of perineal tears	X ^2^ = 12.961	(2)	*p = .002
Suturing of episiotomy	X ^2^ = 2.610	(1)	*p = .042
Manual removal of placenta	X ^2^ = 4.870	(2)	*p = .042

*Note. N* = 85, *p value < .05
